# Two unique cases of Bouveret syndrome with review of literature

**DOI:** 10.1093/jscr/rjac379

**Published:** 2022-08-21

**Authors:** Austin Dixon, Michael D Williams, Kristine Makiewicz, Amna Khokar, Steven Bonomo

**Affiliations:** Department of Surgery, Midwestern University (Chicago College of Osteopathic Medicine), Chicago, IL, USA; Department of General Surgery, Rush University Medical Center, Chicago, IL, USA; Department of General Surgery, Cook County Health (John H. Stroger Hospital), Chicago, IL, USA; Department of General Surgery, Cook County Health (John H. Stroger Hospital), Chicago, IL, USA; Department of General Surgery, Cook County Health (John H. Stroger Hospital), Chicago, IL, USA

## Abstract

Bouveret syndrome is a rare form of gallstone ileus in which a proximally lodged gallstone in the duodenum causes a gastric outlet obstruction. It is a rare condition that can be challenging to manage. Although endoscopic management remains first line, a surgical approach can be needed. We present two cases of Bouveret syndrome. A 65-year-old man with oral squamous cell carcinoma treated with endoscopic management and a 63-year-old woman treated with surgery.

## INTRODUCTION

Bouveret syndrome is a rare form of gallstone ileus in which a proximally lodged gallstone in the duodenum causes a gastric outlet obstruction after traversing a cholecystoduodenal fistula. This syndrome typically presents with epigastric pain, nausea and vomiting in patients with concurrent cholecystitis. Given the nonspecific symptoms, diagnosis is supported with imaging highlighting Rigler’s triad of pneumobilia, gastrointestinal obstruction and ectopic gallstone [1]. Bouveret syndrome is most common in elderly females, with a mean age of 74 and female-to-male sex ratio of 1.86 [2]. Since first described by Beaussier in 1770, little > 300 cases have been published in the literature [3].

Bouveret syndrome can be challenging to manage. Morbidity and mortality are high at 60% and 12–20%, respectively [3]. These high rates of complications are heavily influenced by time to diagnosis and tailored therapeutic approach. Endoscopic management remains first line due to less morbidity and mortality in comparison to surgery [4]. However, success rates of surgery are higher than endoscopy, 78 vs. 29%, respectively [5]. We present two cases of Bouveret syndrome, each with a unique approach specific to the patients’ clinical course, that highlight both endoscopic and surgical treatment strategies.

## CASE REPORT

### Case 1

The first case is a 65-year-old man with oral squamous cell carcinoma. He was treated with chemotherapy and radiation, and subsequently required a tracheostomy and percutaneous endoscopic gastrostomy tube. He had a 2-year history of chronic cholecystitis, though he declined to undergo elective cholecystectomy. He presented to the emergency department with headache, chills and non-bilious emesis following tube feeds. His last bowel movement was 4 days prior, though he was still passing flatus.

**Figure 1:**
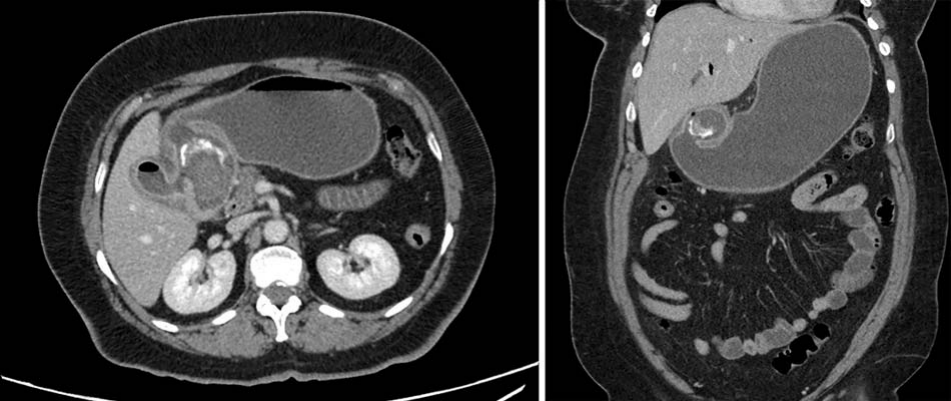
Contrast-enhanced computed tomography (CT). Axial and coronal views of a 2.6 cm stone in the proximal duodenum, gallbladder wall thickening, fat stranding in the porta hepatis and pneumobilia in the non-operative patient.

The patient was afebrile and hemodynamically stable. Physical exam revealed a soft, non-tender abdomen with no distension and no palpable masses. Labs were significant for hyponatremia (130 mmol/L) and leukocytosis (11.6 k/μL). Liver panel was unremarkable. CT of the abdomen with intravenous (IV) contrast demonstrated a 2.6 cm stone in the proximal duodenum, gallbladder wall thickening, fat stranding in the porta hepatis and pneumobilia (Fig. 1).

**Figure 2:**
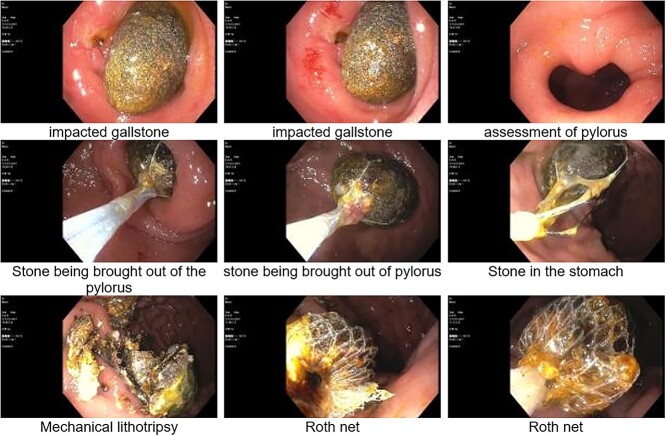
Esophagogastroduodenoscopy (EGD); identification, retrieval and mechanical lithotripsy of a 2 × 3 cm gallstone impacted in the duodenal angle.

The patient was admitted; initial management included gastric decompression, nothing by mouth, fluid resuscitation with IV crystalloid and IV antibiotics for cholecystitis. Gastroenterology was consulted for endoscopic retrieval of the obstructing stone. Esophagogastroduodenoscopy identified a 2 cm by 3 cm gallstone impacted in the duodenal angle (Fig. 2). Just proximal to the impacted stone was a cholecystoduodenal fistula which was freely draining bile. A 3 cm by 6 cm retriever was used to secure the stone, mechanical lithotripsy was performed within the stomach and the fragments were removed through the patient’s mouth. A final endoscopic inspection was performed; no ulceration or necrosis was noted in the duodenum. Patient was discharged home in stable condition 1 day after the procedure with an uneventful postoperative course.

**Figure 3:**
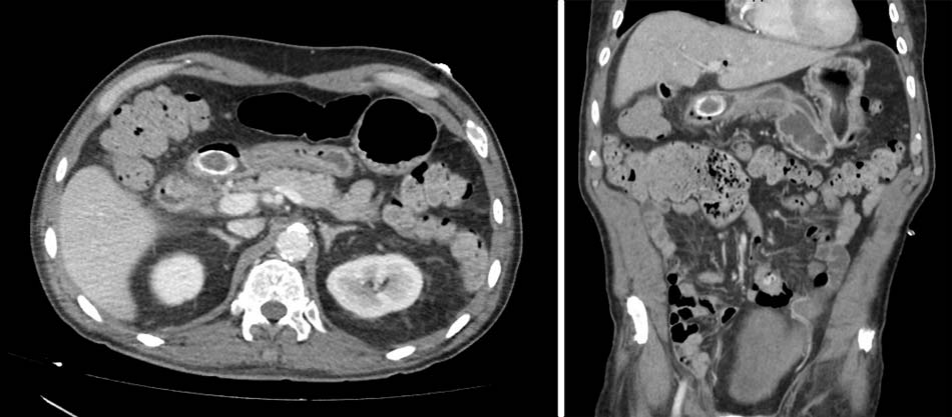
Contrast-enhanced CT. Axial and coronal views of a 4 cm stone in the duodenal bulb, gastric dilation, and pneumobilia in the operative patient.

**Figure 4:**
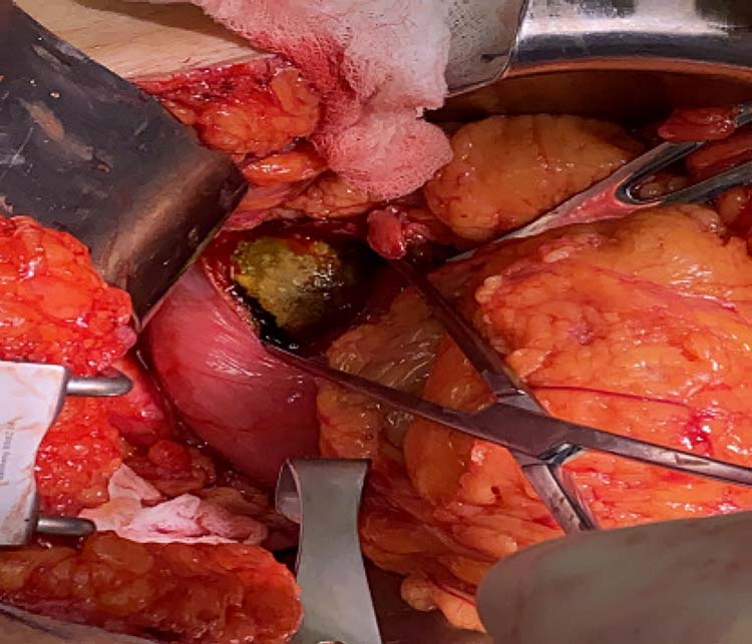
Intraoperative photo; 6 cm × 3 cm gallstone lodged in the duodenal bulb.

### Case 2

A 63-year-old woman with morbid obesity, hypertension and gastritis presented to the emergency department with 2 days of diffuse epigastric pain, nausea and coffee ground emesis. Before onset of these symptoms, the patient described an 8-month history of vague abdominal pain. She was afebrile and hemodynamically stable. Her abdomen was tender to palpation in the epigastrium and right upper quadrant, with no peritoneal signs. Labs were significant for leukocytosis to 17.4 k/μL, lactate to 2.2 mmol/L and Hgb to 10.0 g/dL. All other labs were within normal limits. Abdominal CT demonstrated gallbladder wall thickening, mucosal hyperenhancement and calcified stones within the gallbladder. There was also a 4 cm stone in the duodenal bulb, gastric dilation and pneumobilia. The distended and inflamed gallbladder was inseparable from both the distal stomach and proximal duodenum (Fig. 3).

The patient was admitted and managed with fluid resuscitation and antibiotics for cholecystitis. In addition, gastric decompression, nothing by mouth, pantoprazole and ondansetron were provided for the gastric outlet obstruction with coffee ground emesis. Endoscopic management was discussed with gastroenterology; however, the 4 cm stone was deemed non-retrievable with the available endoscopic equipment and the large size of the stone was concerning for higher perforation risk. Thus, laparotomy was performed to remove the stone. The duodenum was palpated, and the gallstone was identified extending proximally through the pylorus which was dilated. A 6 cm incision was made extending from the pylorus onto normal appearing antrum. A 6 cm by 3 cm stone was extracted using ring forceps (Fig. 4). The gastroduodenostomy was closed transversely in a single layer in an interrupted fashion. A lip of omentum was used to cover the repair. Given the patient’s dense adhesions, a cholecystectomy was not performed at this time.

The patient was diagnosed with pneumonia on post-operative Day 5 and was ultimately discharged on post-operative Day 10. She returned for a 2-week follow-up, where she was healing uneventfully.

## DISCUSSION

Bouveret syndrome can be difficult to manage with its average age of onset at 74 and associated chronic comorbidities such as symptomatic cholecystitis, coronary disease, diabetes mellitus and pulmonary disease [2]. These factors contribute to the high morbidity and mortality at 60% and 12–20%, respectively [3]. In addition, it is extremely rare. Out of all intestinal obstructive cases, gallstone ileus in general accounts for 1–4% of cases. A standard gallstone ileus typically occurs in the terminal ilium, with only 1–3% of obstructions occur proximally in the duodenum as seen with Bouveret syndrome [1]. Given this rarity, there is minimal data to support specific therapy recommendations. Therefore, management should be tailored to the patient at the discretion of the surgeon and the capabilities of the institution. We present two cases which highlight some of the challenges encountered when managing Bouveret syndrome. One case was successfully managed with endoscopic retrieval, whereas the other required surgical approach given endoscopic limitations.

Whenever possible, a multidisciplinary approach is encouraged. Specifically, gastroenterology or other advanced endoscopists should be included in the care of patients with Bouveret syndrome. Endoscopic retrieval is the least invasive method and is considered first line for all patients, but especially in this older population with multiple comorbidities who may not otherwise tolerate the morbidity associated with surgery [4, 6]. Nonetheless, there still exists quite a difference of success rates between endoscopic retrieval and surgical management (29% vs. 78% respectively). Ong et al. highlights this finding and proposes six recommendations that should be taken into consideration when deciding management strategy [5].

When surgery is required for definitive management, it should be approached with caution. Surgical management remains the mainstay of treatment; however, no operative consensus has been reached on surgical techniques in the current literature [7]. Given the presenting complaint, stone removal is of first priority to relieve the obstruction. After retrieval of the impacted gallstone, the question then becomes whether to proceed with a bilioenteric fistula repair and cholecystectomy. As with the rest of this treatment approach, this decision is heavily influenced by the patient’s general condition, age, comorbidities and current inflammatory status. Those in support of forgoing concurrent cholecystectomy highlight that a patent cholecystoduodenal fistula can function as a biliodigestive anastomosis, often spontaneously closes in 30–60 days, and most patients remain asymptomatic after treatment for Bouveret [3]. In addition, in a review of 1001 cases of gallstone ileus in general, only 10% of patients required reoperation with fistula repair due to persistent biliary symptoms [8]. However, if the fistulous tract is obstructed, the gallbladder should be removed to prevent continuation of cholecystitis. In both of our cases, the gallbladder was left in place. In case one, a patent cholecystoduodenal fistula was visualized with endoscopy freely draining bile proximal to the impacted gallstone. A clear fistulous connection between duodenum and gallbladder was present in case two in combination with the fact that the dense adhesion present would have made a potential cholecystectomy complex. In the case of any uncertainty, an endoscopic retrograde cholangiopancreatography (ERCP), cholangiogram or hepatobiliary iminodiacetic acid (HIDA) scan can be considered.

In conclusion, Bouveret syndrome is a rare disease that requires thoughtful and tailored management. Studying Bouveret syndrome in a prospective fashion remains difficult given its rare nature. Therefore, more research is required to determine definitive treatment strategies and surgeons who manage Bouveret syndrome should continue to share their outcomes in the literature.

## CONFLICT OF INTEREST STATEMENT

 None declared.

## FUNDING

This research did not receive any specific grant from funding agencies in the public, commercial, or not-for profit sectors.
